# The clinical decision analysis using decision tree

**DOI:** 10.4178/epih/e2014025

**Published:** 2014-10-30

**Authors:** Jong-Myon Bae

**Affiliations:** Department of Preventive Medicine, Jeju National University School of Medicine, Jeju, Korea

**Keywords:** Decision analysis, Decision-making, Decision support techniques, Evidence-base medicine

## Abstract

The clinical decision analysis (CDA) has used to overcome complexity and uncertainty in medical problems. The CDA is a tool allowing decision-makers to apply evidence-based medicine to make objective clinical decisions when faced with complex situations. The usefulness and limitation including six steps in conducting CDA were reviewed. The application of CDA results should be done under shared decision with patients’ value.

## INTRODUCTION

Clinical practice, aimed at solving clinical problems of individual patients, is an act of continual decision-making. The best clinical decision refers to making a choice that maximizes effectiveness and minimizes harm [1]. Nevertheless, when the supporting evidences were scant, decision-making depends on the subjective intuition of the physician and then becomes unpredictable and non-reproducible [2].

Around the 1990s, Evidence-based Medicine (EBM), which was suggested as a methodology for making clinical decisions based on the best evidence [3-6], expanded across the entire field of healthcare, and the terminology “evidence-based decision-making” was introduced [7-9]. By overcoming the complexity of medical environment [10-13] and the uncertainty of clinical decisions [14-17], the EBM aims to pursue qualitative improvements in healthcare [18-21]. Because clinical decisions are also directly related to the development and expansion of clinical treatment guidelines, approval of new drugs, drug prescriptions, the applicability of medical insurance for procedures, and healthcare policies [22,23].

McCreery & Truelove [20] summarized five methodologies for decision-making: (1) Bayes’ theorem, (2) decision-tree design, (3) receiver-operating-characteristic curves, (4) sensitivity analysis, (5) utilities assessment. The clinical decision analysis (CDA) was suggested to make a clinical decision based on objectively quantitative indices calculated by using these methodologies [1]. This manuscript aims at reviewing the CDA methodology by definition, process, usefulness, and limitations.

## BODY

### Definition of clinical decision analysis

In 1976, Bear & Schneiderman [24] suggested the terminology “clinical decision analysis” with the intention of applying the concept of decision analysis (DA), which had already been used in business and other social sciences, to the field of clinical practice. In order to understand the meaning of the term CDA, it is necessary to also look at the term DA coined by Raiffa [25] in 1968. In Appendix 1, paragraphs defining DA and CDA were arranged chronologically. CDA can be seen as a way of overcoming ‘uncertainty’ .

### Process of clinical decision analysis

**Figure 1.**
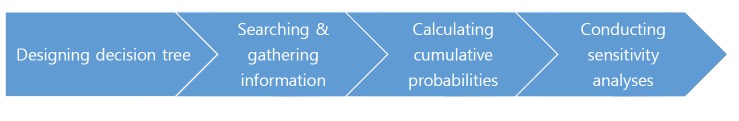
Steps of clinical decision analysis using decision tree method.

Watts [26] proposed that CDA should consist of six stages including cost analysis, whereas Sackett et al. [27] proposed six stages including clinical practice. These process was well explained in the articles of Korah et al. [28] and Aleem et al. [1]. Depending on the methodology used, the CDA stages can be summarized as follows: (1) designing a decision tree showing all instances that can occur in a particular situation, (2) securing the likelihood and outcome utility values for each instance by conducting a literature search, (3) calculating the probabilities of cumulative expectation using the Bayesian theorem, and (4) performing a sensitivity analysis and assessing the variables needed for clinical decision-making (Figure 1).

Since the content of the series of tasks that must be performed (including the construction of the decision tree) varies depending on the research questions [29], reference papers for different research questions are presented in Appendix 2. A detailed explanation is not included. Instead, the significance of performing a sensitivity analysis in the final stage will be discussed. The cumulative expectation probabilities obtained by using a decision tree vary according to the input values of outcome utility and likelihood [30]. Consequently, by estimating the vulnerability (how much the outcomes change according to fluctuations in the input values) the ultimate objective was to reduce uncertainty in decision-making [31]. In addition, sensitivity analysis could be used to elucidate the extent to which a given clinical situational variable affects the decision [28,32-34], so that these variables can be used as latent predictor variables for clinical prediction rules (CPR) [35-38]. Moreover, areas requiring future clinical research can be identified [39], and logical systematic errors in the designed decision tree can be debugged [30]. Traditional n-way sensitivity analysis [39,40] has been used as the statistical method for conducting a sensitivity analysis, but more recently, the Markov Chain Monte Carlo simulation methods [39,41-43] has been mainly used.

In the CDA process, the most difficult stages are the design of the decision tree [1,40,44-46], the debugging of logical errors in the designed tree [30], the calculation of the cumulative probability, and the Monte Carlo simulation for the sensitivity analysis [47]. The recent development of the commercial software TreeAge Pro [48] is making these processes easier, and the importance of the literature search to find the appropriate values for analysis is being emphasized [1,49]. The latter is crucial since the meaning of the relevant values varies by country and generation [50,51].

### Usefulness of clinical decision analysis

The usefulness of CDA in a clinical setting, being performed with the aim of overcoming complexity and uncertainty in clinical decisions, can be broadly summarized into three types.

First, true to its original purpose, CDA provides the decision maker with objective evidence to make a judgment [1,52-55]. Consistent and reproducible decision-making can reduce the misuse of medical resources caused by uncertainty, improve the patient-physician relationship [18,30,56], and lead to qualitative improvements in healthcare [57,58].

Second, CDA reveals the environmental variables that need to be considered when making a decision in a clinical setting [49,59,60]. This can be useful in decision-making not only for physicians, but also for medical insurance companies or decision-makers in healthcare administration [32,33,61].

Third, by elucidating these predictor variables, the sensitivity analysis can be useful for CPR development, and can be applied to utility analysis and even cost analysis, as it reveals cases of insufficient evidence [5,26,33,60,62,63].

### Limitations of clinical decision analysis

It has been claimed that the results obtained by CDA using a decision tree should only be used as a reference in decision-making, and are not guaranteed as absolute [45,64]. This implies that the CDA methodology has several limitations. Since CDA was proposed in 1970, doubts have been raised about its usefulness [57]. Aleem et al. [45] have presented a summary of these limitations from the perspective of a performance and of outcome analysis: (1) simplification errors may occur when measuring the final outcome of treatment decisions with indices such as quality-adjusted life years (QALYs) [1,32,49]; (2) performing a time-consuming analysis adequately in a busy clinical environment is difficult [45]; and (3) various factors (including potential harmful, expense, and patient preference for medical services) are involved in decision-making, and these cannot be accurately reflected in a decision tree [63,65]. Therefore, even if a CDA result is available, diverse decision-making is ultimately inevitable [10,66,67].

## CONCLUSION AND PROPOSALS

Although the nature of medical treatment makes diverse decision-making inevitable, Black et al. [68] nevertheless emphasize the usefulness of CDA based on its ability to overcome uncertainty in medical treatment, and because the patient will encounter objective evidence through the internet or other sources as part of the shared decisions involving the patient. Considering the trends, four suggestions would be prepared to stimulate related research in South Korea:

First, detailed care is required when interpreting the results of CDA studies and applying them to clinical practice. According to the standards for evaluating the validity and applicability of content from CDA papers [55,69-71], not only does an appropriate evaluation need to be performed, but the predicted likelihoods as the outcomes of the analysis must also be applied to a patient group rather than to individual patients [32]. In addition, data from persons of different nationalities used for CDA have different meanings [51]. In this regard, it is necessary to conduct related studies to gain data on Korean individuals. If CDA is performed, efforts to adhere to the seven principles demanded by Lurie & Sox [72] must be made.

Second, the patients’ values must be reflected in decision-making. CDA relates to the combined value of the available evidences, but EBM [73-75] emphasizes that the manner in which this evidence is interpreted and reflected in the decision depends on the experiences of the clinical team [76] and the preferences of the patient [77]. In order to synthesize these three factors, Straus [78] proposed the likelihood of being helped or harmed index. In this way, shared decisions [79,80], meaning that decisions made together with the patient, are increasingly being demanded nowadays, and there is an emphasis on patient-centered clinical service [81,82]. This is in line with the principles of medical ethics [83-85] and can achieve the goal of restricting uncertainty in clinical treatment [14,86,87].

Third, since the patient’s opinion should be positively reflected in the decision-making, decision aids for patients should be developed [88-90] in addition to increased CDA research. As seen in the various examples of decision aids described by O’Connor [91], decision aids are instruments that help making a value-based decision in accordance with the patient’s individual preferences but are different from educational material for patients [92]. Given that research has consistently shown that these instruments are helpful for patients [93-95], more decision aids for Korean patients must be developed.

Fourth, CDA using decision trees, as dealt with in this manuscript, became active in the 1990s, and entering the 2000s various methodologies have been developed and are being applied to healthcare and medical treatment, such as supporting vector machines [96], random forest [97], and deep learning [98]. In the future, these methodologies must be investigated, so that they may be applied to various areas in healthcare with an understanding of their strengths and weaknesses.

## Appendix 1. Summary tables of the definition-related sentences about decision analysis (DA) and clinical decision analysis (CDA)

**Table:**

	Sources references	Related sentences
DA	A01	DA -an explicit, normative and analytic approach to making decisions under uncertainty- provides a probabilistic framework for exploring difficult problems in non-deterministic domains.
	A02	DA is the application of explicit, quantitative methods to analyze decisions under conditions of uncertainty.
	A03	DA formalizes the decision process, highlights the factors that influence the decision, and applies mathematical rigour to quantify decision-making.
CDA	A04	CDA seeks to identify the optimal management strategy by modelling the uncertainty and risks entailed in the diagnosis, natural history, and treatment of a particular problem or disorder.
	A05	CDA is a systematic method for making wise choices under just such circumstances.
	A06	(C)DA in a quantitative approach for dealing with the uncertainties inherent in many medical decisions, including decisions about genetic testing.
	A07	(C)DA is a quantitative by an ever increasing number of costly and confusing application of probability and utility theory to decision diagnostic tests and therapeutic interventions, decision-making under conditions of uncertainty.
	A08	(C)DA is a quantitative approach to decision-making under conditions of uncertainty that can be applied to specific types of clinical problems.
	A09	CDA is a process whereby different treatment options are assessed systematically.
	A10	(C)DA is a formal, mathematical approach to analyzing difficult decisions faced by clinical decision makers (i.e. patients, clinicians, policy-makers).
	A11	(C)DA is a formal, quantitative method for systematically comparing the benefits and harms of alternative clinical strategies under circumstances of uncertainty.
	A12	(C)DA is a tool that allows users to apply evidence-based medicine to make informed and objective clinical decisions when faced with complex situations.
	A13	(C)DA is a simulation, model-based research technique in which investigators combine information from a variety of sources to create a mathematical model representing a clinical decision.
	A14	CDA - the application of DA to a clinical or patient-based setting - is a technique that incorporates literature-derived probabilities with expert and patient preferences to result in an informed clinical decision.

## Appendix 2. Articles referring to conducting clinical decision analysis using decision tree method

**Table:**

Epidemiology	Domain	Related publications
Basic	Threshold	B01, B02
	Prognostic factor	B03
	Cost factor	B04
	Genetic counseling	B05
Clinical	Screening test	B06
	Diagnostic test	B07, B08
	Procedures	B09-B13
	Drug	B14, B15
