# Supplementary material for: The clinical decision analysis using decision tree
Source: Epidemiol Health. 2014 Oct 30;36:e2014025. doi: 10.4178/epih/e2014025 (PMC4251295; doi:10.4178/epih/e2014025)
Supplement: Supplementary file 1 [file epih-36-e2014025-supplementary.pdf]

<연구방법론>

임상결정분석 – 결정나무 중심으로

Clinical Decision Analysis using Decision Tree

Abstract

The clinical decision analysis (CDA) has used to overcome complexity and uncertainty in medical problems. The CDA is a tool allowing decision-makers to apply evidence-based medicine to make objective clinical decisions when faced with complex situations. The usefulness and limitation including six steps in conducting CDA were reviewed. The application of CDA results should be done under shared decision with patients' value.

Key words: Decision Analysis, Decision making, Decision support techniques, Evidence-base medicine

1. 서론

개별 환자의 임상적 문제점들을 해결하기 위한 임상진료 (clinical practice)는 의사결정 (decision making)의 연속적 행위이며, 최선의 임상 결정 (clinical decision)이란 다양한 선택 중에서 효과 (Effectiveness)를 최대화하고 유해 (Harm)를 최소화할 수 있는 것을 선택하는 것이다 [1]. 그러나 이를 뒷받침해주는 근거들이 부족한 경우에는 주치의의 주관적 직감 (intuition)에 의존하여 예측불가하고 재현불가한 의사결정을 하게 된다 [2].

1990년대 전후로 최선의 근거에 따라 임상결정을 하려는 방법론으로 제기된 근거중심의학 (Evidence-based Medicine, 이하 EBM)이 [3-6] 보건의료영역 전반에 확산되면서, 근거중심의사결정 (Evidence-based decision making)이란 용어가 제기되었다 [7-9]. 이처럼 진료결정에 있어 이용 가능한 최선의 근거들을 적극 활용하자는 주장의 배경에는, 임상결정을 위해서는 의료 환경의 복잡성 (complexity) [10-13]과 의료 결정의 불확실성 (uncertainty) [14-17]을 극복하여 보건의료의 질적 향상을 도모하기 위함이다 [18-21]. 다시 말해서, 임상결정은 단지 환자진료 영역에만 국한되는 것이 아니라 임상진료지침 개발 및 확산, 의약품의 승인 및 허가, 약물 처방 및 시술의 의료보험 적용, 보건의료 정책 등과도 직결되기 때문이다 [22,23].

McCreery & Truelove [20]는 의사결정을 위하여 5가지 방법론 - ① Bayes' theorem ② decision-tree design ③ receiver-operating-characteristic curves (ROC) ④ sensitivity analysis ⑤ utilities assessment –이 활용되어 왔다고 정리하였다. 이들을 최대한 적용해서 객관적이고 계량적인 지표를 산출하고, 이를 의사결정에 활용하려는 연구방법론으로 임상결정분석 (clinical decision analysis, 이하 CDA)이라 부르게 되었다 [1]. 본 원고는 McCreery & Truelove [20]이 제시한 CDA에 관하여 정의, 수행과정, 유용성 및 한계점의 4 부분으로 나

누어 살펴보고자 한다.

2. 본론

가. 임상결정분석 (CDA)의 정의

1976년 Bear & Schneiderman [24]은 이미 경영 등의 사회과학 영역에서 활용되고 있던 decision analysis (DA) 란 개념을 임상영역에 적용하자는 의미로 'clinical decision analysis'란 용어를 제안하였다. 따라서 CDA란 용어의 의미를 알기 위해서는 1968년 Raiffa [25]가 제창한 DA 란 용어를 따로 살펴볼 필요가 있겠다. 부록 1은 CDA와 관련한 논문들의 본문에서 DA 와 CDA의 정의를 제시한 구절을 따로 떼어서 연도별로 정리한 것이다. 부록 1에서 CDA는 'uncertainty'를 극복하기 위한 하나의 방편임을 확인할 수 있다.

나. 임상결정분석의 수행단계

CDA 수행과정에 있어서 Watts (1989) [26]는 비용분석을 포함한 6 단계를 제시하였다. 그러나 Sackett et al. [27]는 임상실무를 반영하여 비용분석을 제외한 6 단계를 제시하였고, 그 실제 과정을 Korah et al. (1999) [28]과 Aleem et al. (2008) [1]의 관련 논문에서 잘 설명하고 있다. 적용하는 방법론에 따라 수행 단계를 요약하자면 ① 특정상황에서 일어날 모든 경우를 나타내는 결정구조 (decision tree)를 설계한 다음 ② 문헌탐색으로 각 경우의 발생 확률과, 결과의 편익 (utility) 값을 확보하고 ③ Bayesian theorem을 적용하여 누적예상 수준을 산출한다. 마지막으로 ④ 민감도 분석 (sensitivity analysis)을 해서 의사결정에 유의해야할 변수들을 살펴본다 (Fig 1).

결정구조 설계를 포함한 일련의 수행내용은 연구 질문별로 달라지므로 [29], 부록 2에서 연구 질문별로 참조할 논문들을 제시하고, 자세한 설명은 생략한다. 대신 마지막 단계에서 민감도 분석을 수행하는 의미를 알아보고자 한다. 결정구조를 이용하여 얻어낸 누적예상 수준은 발생 확률과 결과 편익의 입력 값에 따라 달라진다 [30]. 따라서 입력 값의 변동에 따라 결과 수준이 얼마나 변화하느냐의 취약성 (vulnerability)을 가늠하는 것으로 [1], 궁극적으로는 의사 결정의 불확실성을 통제하려는 목적을 가졌다 [31]. 또한 민감도 분석을 통해 임상의 어떤 상황변수가 결정에 얼마만큼의 영향을 미치는가를 알아낼 수 있어서 [28, 32-34], 바로 clinical prediction rules (이하 CPR) [35-38]의 잠재적인 예측 변수 (predictor variables)로 활용할 수 있다. 이외에도 향후 임상연구가 필요한 부분을 찾아낼 수 있게 해주며 [39], 설계한 결정 구조에서 논리체계상의 오류를 수정할 (debugging) 기회를 얻기도 한다 [30]. 민감도 분석에 사용하는 통계법으로 traditional n-way sensitivity analysis [39,40]를 적용해 왔으나, 지금은 Markov Chain Monte Carlo simulation methods [39,41-43]를 주로 사용하고 있다.

이상의 CDA 수행 과정에서 결정구조를 설계하고 [1,40,44-46], 설계한 구조에서의 논리적 오류를 찾아 수정 (debugging)하는 것 [30]과 누적발생 수준 산출과 민감도 분석을 위한 Monte Carlo Simulation을 수행하는 것이 가장 까다로운 단계이다 [47]. 그러나 최근에 TreeAge Pro 란 상용프로그램[48]이 개발되어서 이 과정들을 용이하게 수행할 수 있게 되면서, 이제는 분석에 요구되는 적절한 수치들을 찾아내는 문헌검색의 중요성이 더 강조되고 있다 [1,49]. 해당 값들의 의미는 나라별, 시대별로 달라지기 때문이다 [50,51].

다. 임상결정분석의 유용성

의료결정의 복잡성과 불확실성을 극복하려는 배경에서 수행되어 온 CDA가 임상현장에서 갖는 유용성은 크게 3가지로 정리해 볼 수 있다.

첫째, 본래의 목적처럼 의사결정자에게 판단의 객관적 근거를 제시해 준다는 것이다 [1,52-55]. 일관성과 재현가능성을 가진 의사결정은 곧 의료의 불확실성으로 생기는 의료자원의 오남용을 줄이고, 환자-의사 관계를 개선시켜 주면서 [18,30,56], 보건의료 전반의 질적 향상을 도모할 수 있게 한다 [57,58].

둘째, 임상 현장에서 의사결정에 의미있게 고려해야 할 환경변수가 무엇인가를 알게 해준다 [49,59,60]. 이는 곧 진료의사뿐만 아니라, 의료보험회사나 보건행정 결정자의 의사결정에도 유용하게 활용될 수 있게 해준다 [32,33,61].

셋째, 민감도 분석에서 언급했듯이 예측변수를 알게 해주어서 CPR 개발에 활용할 뿐만 아니라, 부족한 근거를 알려주어서 편익분석 (utility analysis), 나아가 비용분석 (cost analysis)까지 활성화시킬 수 있게 한다 [5,26,33,60,62,63].

라. 임상결정분석의 한계점

Decision Tree 를 이용한 CDA 수행으로 얻어낸 결과는 의사결정에 절대적인 것으로 보장받지 못하며 단지 참고자료로 활용해야 한다는 주장[45,64] 이 있다는 것은 CDA가 여러 측면에서 한계를 가지고 있다는 뜻이다. 실제로 CDA 가 제기된 1970년부터 CDA의 유용성에 대한 의문이 제기되어 왔다 [57]. 이들 한계점을 Aleem et al. [45]은 수행과정 측면과 결과해석 측면에서 정리하여 제시하였다.

이들을 요약을 하자면 (1) 진료결정의 최종 결과를 의미하는 편익 (utilitiy)을

quality-adjusted life years (QALYs) 등의 지표로 계량화하는 과정에서 단순화의 오류가 생기며 [1,32,49] (2) 바쁜 진료환경에서 시간이 소모되는 분석을 제대로 해 내기가 어려우며 [45] (3) 의사결정에는 의료서비스의 유해성, 비용, 환자 선호도 등의 다양한 요소들이 개입되는데, 이들을 결정구조에 제대로 반영할 수 없다는 것이다 [63,65]. 이에 따라 CDA 분석 결과가 있음에도 불구하고 결국은 다양한 의사결정이 이루어 질 수밖에 없다는 것이다 [10,66,67].

3. 결어 및 제언

의료의 특성상 다양한 결정이 이루어질 수밖에 없다는 결론에 대하여, Black et al. [68]은 그럼에도 불구하고 의료의 불확실성을 극복할 수 있으며, 환자를 참여시키는 결정(shared decision)과정에서 환자는 인터넷 등에서 객관적인 근거들을 찾게 된다는 이유 등을 들어서 CDA의 유용성을 강조하고 있다. 이런 흐름 속에서 국내 관련 연구의 활성화를 위하여 4가지로 정리하여 제언코자 한다.

첫째, CDA 분석연구의 결과를 해석하고 임상에 적용하는 과정에 세심한 주의가 필요하다. CDA 논문의 내용 타당성과 적용가능성을 평가하는 기준 [55, 69-71]에 따라 적절한 평가가 이루어 져야 할 뿐만 아니라, 분석결과인 예상발생수준은 개별환자가 아니라 환자군에 적용되는 것이라는 점[32]이다. 또한 CDA에 반영한 자료원이 자국민이 아니라면 다른 의미를 가질 수 있다는 점 [51]에서 우리 국민을 대상으로 한 근거 자료 생성을 위한 관련 연구가 꾸준히 이루어 져야 한다. 그리고 CDA를 수행한다면 Lurie & Sox [72]가 요구하는 7가지 사항을 제대로 지키도록 노력해야 한다.

둘째, 의사결정에는 환자의 가치가 반영되어야 한다. CDA는 가용한 근거들의 종합치에 해당하며, 이를 어떻게 해석하고 결정에 반영할 것인가는 의료진의 경험 [73]과 환자의 선호도 [74]에 달렸다고 EBM은 강조한다 [75-77]. 이 3가지 요소들을 합성하기 위해 Straus [78]는 the Likelihood of being helped or harmed (LHH) 지표를 제시하였다. 이처럼 환자와 같이 의사결정을 한다는 shared decision [79,80]은 오늘날 환자중심 (patient-centered)의 의료서비스가 강조되는 상황에서 더욱 요구되고 있다 [81,82]. 이는 의료윤리의 원칙에도 부합하며 [83-85], 의료의 불확실성을 통제하는 목적을 달성할 수 있는

것이다 [14,86,87].

셋째, 의사결정에 환자의 의견을 적극 반영하려면 CDA 연구 활성화와 함께, 환자용 결정조력도구 (decision aids)의 개발도 이루어져야 한다 [88-90]. O'connor [91]는 결정 조력도구의 각종 예들을 보여주고 있듯이, 결정조력도구는 환자 교육홍보 자료와 다르며, 환자 개인의 선호도에 맞추어 가치바탕결정 (value-based decision)을 하는데 도움을 주는 도구이다 [92]. 이들 도구는 환자에게 도움이 된다는 연구결과가 일관성있게 제시되는 만큼 [93-95], 국내 환자용 도구 개발이 활성화되어야 한다.

넷째, 본 원고에서 다룬 Decision tree 를 이용한 의사결정분석은 1990년대에 활발히 이루어 진 것으로, 2000년대를 들어서는 supporting vector machine [96], random forest [97], deep learning [98] 등의 다양한 방법론이 개발되어 보건의료 연구에 적용되고 있다. 이들 방법론의 장단점을 이해하면서 다양한 보건의료 영역에서 적용할 수 있도록 방법론 고찰이 추후에 이루어 져야 한다.

감사의 글

이 논문은 2014학년도 제주대학교 학술진흥연구비 지원사업에 의하여 연구되었음

References

1. Aleem IS, Schemitsch EH, Hanson BP. What is a clinical decision analysis study? Indian J Orthop 2008;42:137-139.
2. Banning M. A review of clinical decision making: models and current research. J Clin Nurs 2008;17:187-195.
3. Rosenberg WM, Sackett DL. On the need for evidence-based medicine. Therapie 1996;51:212-217.
4. Sackett DL. Evidence-based medicine. Spine 1998;23:1085-1086.
5. Djulbegovic B, Hozo I, Lyman GH. Linking evidence-based medicine therapeutic summary measures to clinical decision analysis. MedGenMed 20002:E6.
6. Bae JM, Park BJ, Ahn YO. Perspectives of clinical epidemiology in Korea. J Korean Med Assoc 2013;56:718-723. (korean)
7. Dowie J. 'Evidence-based', 'cost-effective' and 'preference-driven' medicine: decision analysis based medical decision making is the pre-requisite. J Health Serv Res Policy 1996;1:104-113.
8. Teutsch SM, Berger ML. Evidence synthesis and evidence-based decision making: related but distinct processes. Med Decis Making 2005;25:487-489.
9. Bates DW, Kuperman GJ, Wang S, Gandhi T, Kittler A, Volk L, et al. Ten commandments for effective clinical decision support: making the practice of evidence-based medicine a reality. J Am Med Inform Assoc 2003;10:523-530.
10. Balla JI, Elstein AS, Christensen C. Obstacles to acceptance of clinical decision analysis. BMJ 1989;298:579-582.
11. Hamilton JD. Do we under utilise actuarial judgement and decision analysis? Evid Based Ment Health 2001;4:102-103.
12. Galanter CA, Patel VL. Medical decision making: a selective review for child psychiatrists and psychologists. J Child Psychol Psychiatry 2005;46:675-689.
13. Thompson C, Cullum N, McCaughan D, Sheldon T, Raynor P. Nurses, information use, and clinical decision making--the real world potential for evidence-based decisions in nursing. Evid Based Nurs 2004;7:68-72.
14. Beresford EB. Uncertainty and the shaping of medical decisions. Hastings Cent Rep 1991;21:6-11.
15. West AF, West RR. Clinical decision-making: coping with uncertainty. Postgrad Med J 2002;78:319-321.
16. Hu W, Kemp A, Kerridge I. Making clinical decisions when the stakes are high and the evidence unclear. BMJ 2004;329:852-854.
17. Thornton JG, Lilford RJ, Johnson N. Decision analysis in medicine. BMJ 1992;304:1099-1103.

18. Bae JM. Academic strategies based on evidence-practice gap. Hanyang Medical Reviews 2014. (in press)

19. Brazil K, Ozer E, Cloutier MM, Levine R, Stryer D. From theory to practice: improving the impact of health services research. BMC Health Serv Res 2005;5:1.

20. McCreery AM, Truelove E. Decision making in dentistry. Part I: A historical and methodological overview. J Prosthet Dent 1991;65:447-451.

21. Myers J, McCabe SJ. Understanding medical decision making in hand surgery. Clin Plast Surg 2005;32:453-461.

22. Atkins D. Creating and synthesizing evidence with decision makers in mind: integrating evidence from clinical trials and other study designs. Med Care 2007;45(10 Supl 2):S16-S22.

23. Garrison LP Jr, Neumann PJ, Erickson P, Marshall D, Mullins CD. Using real-world data for coverage and payment decisions: the ISPOR Real-World Data Task Force report. Value Health 2007;10:326-335.

24. Bear R, Schneiderman J. Decision analysis in clinical medicine. Can Med Assoc J 1976;115:833-836.

25. Raiffa H. Decision analysis: Introductory lectures on choices under uncertainty. MA: Addision-Wesley Publishing Co.;1968.

26. Watts NT. Clinical decision analysis. Phys Ther 1989;69:569-576.

27. Sackett DL, Haynes RB, Guyatt GH, Tugwell P. Clinical epidemiology: a basic science for clinical medicine. 2ne ed. Toronto: Little, Brown & Co.; 1991.p.140-145.

28. Korah S, Thomas R, Muliyil J. An introduction to clinical decision analysis in ophthalmology. Indian J Ophthalmol 1999;47:41-48.

29. Lilford RJ, Pauker SG, Braunholtz DA, Chard J. Decision analysis and the implementation of research findings. BMJ 1998;317:405-409.

30. Krahn MD, Naglie G, Naimark D, Redelmeier DA, Detsky AS. Primer on medical decision analysis: Part 4--Analyzing the model and interpreting the results. Med Decis Making 1997;17:142-151.

31. Briggs AH. Handling uncertainty in cost-effectiveness models. Pharmacoeconomics 2000;17:479-500.

32. van der Velde G. Clinical decision analysis: an alternate, rigorous approach to making clinical decisions and developing treatment recommendations. J Can Chiropr Assoc 2005;49:258-263.

33. Graham B, Detsky AS. The application of decision analysis to the surgical treatment of early osteoarthritis of the wrist. J Bone Joint Surg Br 2001;83:650-654.

34. Hirai S, Ono J, Odaki M, Serizawa T, Sato M, Isobe K, et al. Treatment of asymptomatic unruptured intracranial aneurysms. A clinical decision analysis. Interv Neuroradiol 2001;7(Suppl 1):61-64.

35. Huijbregts P. Clinical prediction rules: time to sacrifice the holy cow of specificity? J Man Manip Ther 2007;15;5-8.

36. Cook CE. Potential pitfalls of clinical prediction rules. J Man Manip Ther 2008;16:69-71.

37. Ingui BJ, Rogers MA. Searching for clinical prediction rules in MEDLINE. J Am Med Inform Assoc 2001;8:391-397.

38. Vickers AJ, Cronin AM. Traditional statistical methods for evaluating prediction models are uninformative as to clinical value: towards a decision analytic framework. Semin Oncol 2010;37:31-38.

39. Aoki N, Beck JR, Kitahara T, Ohbu S, Soma K, Ohwada T, et al. Reanalysis of unruptured intracranial aneurysm management: effect of a new international study on the threshold probabilities. Med Decis Making 2001;21:87-96.

40. Hagen MD. Decision analysis: a review. Fam Med 1992;24:349-354.

41. Minelli C, Abrams KR, Sutton AJ, Cooper NJ. Benefits and harms associated with hormone replacement therapy: clinical decision analysis. BMJ 2004;328:371.

42. Naimark D, Krahn MD, Naglie G, Redelmeier DA, Detsky AS. Primer on medical decision analysis: Part 5--Working with Markov processes. Med Decis Making 1997;17:152-159.

43. Doubilet P, Begg CB, Weinstein MC, Braun P, McNeil BJ. Probabilistic sensitivity analysis using Monte Carlo simulation. A practical approach. Med Decis Making 1985;5:157-177.

44. Podgorelec V, Kokol P, Stiglic B, Rozman I. Decision trees: an overview and their use in medicine. J Med Syst 2002;26:445-463.

45. Aleem IS, Jalal H, Aleem IS, Sheikh AA, Bhandari M. Clinical decision analysis: Incorporating the evidence with patient preferences. Patient Prefer Adherence 2009;3:21-24.

46. Detsky AS, Naglie G, Krahn MD, Redelmeier DA, Naimark D. Primer on medical decision analysis: Part 2—Building a tree. Med Decis Making 1997;17:126-135.

47. Doan A, Haddawy P, Kahn CE Jr. Decision-theoretic refinement planning: a new method for clinical decision analysis. Proc Annu Symp Comput Appl Med Care 1995:299-303.

48. TreeAge Software Inc. TreeAge Pro Healthcare [cited 2014 Sep 9]. Available from: https://www.treeage.com/shop/treeage-pro-healthcare/

49. Naglie G, Krahn MD, Naimark D, Redelmeier DA, Detsky AS. Primer on medical decision analysis: Part 3--Estimating probabilities and utilities. Med Decis Making 1997;17:136-141.

50. Torrance GW. Utility approach to measuring health-related quality of life. J Chronic Dis 1987;40:593-603.

51. Clancy CM, Cronin K. Evidence-based decision making: global evidence, local decisions. Health Aff. 2005;24:151-162.

52. Sarasin FP. Decision analysis and its application in clinical medicine. Eur J Obstet Gynecol Reprod Biol

2001;94:172-179.

53. Yentis SM. Decision analysis in anaesthesia: a tool for developing and analysing clinical management plans. Anaesthesia 2006;61:651-658.

54. Sisson JC, Schoomaker EB, Ross JC. Clinical decision analysis. The hazard of using additional data. JAMA 1976;236:1259-1263.

55. Richardson WS, Detsky AS. Users' guides to the medical literature. VII. How to use a clinical decision analysis. A. Are the results of the study valid? Evidence-Based Medicine Working Group. JAMA 1995;273:1292-1295.

56. Kassirer JP, Moskowitz AJ, Lau J, Pauker SG. Decision analysis: a progress report. Ann Intern Med 1987;106:275-291.

57. Dolan JG. Clinical decision analysis. Med Decis Making 2001;21:150-151.

58. Pauker SG, Kassirer JP. Decision analysis. N Engl J Med 1987;316:250-258.

59. Stockstill JW, Bowley JF, Attanasio R. Clinical decision analysis in fixed prosthodontics. Dent Clin North Am 1992;36:569-580.

60. McCreery AM, Truelove E. Decision making in dentistry. Part II: Clinical applications of decision methods. J Prosthet Dent 1991;65:575-585.

61. Burd RS, Sonnenberg FA. Decision analysis: a basic overview for the pediatric surgeon. Semin Pediatr Surg 2002;11:46-54.

62. Burford B, Lewin S, Welch V, Rehfuess E, Waters E. Assessing the applicability of findings in systematic reviews of complex interventions can enhance the utility of reviews for decision making. J Clin Epidemiol 2013;66:1251-1261.

63. Burch J, Hinde S, Palmer S, Beyer F, Minton J, Marson A, et al. The clinical effectiveness and cost-effectiveness of technologies used to visualise the seizure focus in people with refractory epilepsy being considered for surgery: a systematic review and decision-analytical model. Health Technol Assess 2012;16:1-157.

64. Buchanan JG. An introduction to clinical decision analysis: bone marrow transplantation for aplastic anemia. Aust N Z J Med 1983;13:451-456.

65. Bhandari M, Devereaux PJ, Swiontkowski MF, Tornetta P 3rd, Obremskey W, Koval KJ, et al. Internal fixation compared with arthroplasty for displaced fractures of the femoral neck. A meta-analysis. J Bone Joint Surg Am 2003;85-A(9):1673-1681.

66. Brookhart MA, Solomon DH, Wang P, Glynn RJ, Avorn J, Schneeweiss S. Explained variation in a model of therapeutic decision making is partitioned across patient, physician, and clinic factors. J Clin Epidemiol 2006;59:18-25.

67. Sekimoto M, Imanaka Y, Kitano N, Ishizaki T, Takahashi O. Why are physicians not persuaded by

scientific evidence? A grounded theory interview study. BMC Health Serv Res 2006;6:92.

68. Blank T, Graves K, Sepucha K, Llewellyn-Thomas H. Understanding treatment decision making: contexts, commonalities, complexities, and challenges. Ann Behav Med 2006;32:211-217.

69. Richardson WS1, Detsky AS. Users' guides to the medical literature. VII. How to use a clinical decision analysis. A. Are the results of the study valid? Evidence-Based Medicine Working Group. JAMA 1995;273:1292-1295.

70. Richardson WS, Detsky AS. Users' guides to the medical literature. VII. How to use a clinical decision analysis. B. What are the results and will they help me in caring for my patients? Evidence Based Medicine Working Group. JAMA 1995;273:1610-1613.

71. Thornhill J, Judd M, Clements D. CHSRF knowledge transfer: (re)introducing the self-assessment tool that is helping decision-makers assess their organization's capacity to use research. Healthc Q 2009;12:22-24.

72. Lurie JD, Sox HC. Principles of medical decision making. Spine. 1999;24:493-498.

73. Dans AL, Dans LF, Guyatt GH, Richardson S. Users' guides to the medical literature: XIV. How to decide on the applicability of clinical trial results to your patient. Evidence-Based Medicine Working Group. JAMA 1998;279:545-549.

74. Goetghebeur MM, Wagner M, Khoury H, Levitt RJ, Erickson LJ, Rindress D. Evidence and Value: Impact on DEcisionMaking--the EVIDEM framework and potential applications. BMC Health Serv Res 2008;8:270.

75. Sackett DL, Rosenberg WM, Gray JA, Haynes RB, Richardson WS. Evidence based medicine: what it is and what it isn't. BMJ 1996;312:71-72.

76. Barratt A. Evidence Based Medicine and Shared Decision Making: the challenge of getting both evidence and preferences into health care. Patient Educ Couns 2008;73:407-412.

77. Tilburt JC. Evidence-based medicine beyond the bedside: keeping an eye on context. J Eval Clin Pract 2008;14:721-725.

78. Straus SE. Individualizing treatment decisions. The likelihood of being helped or harmed. Eval Health Prof 2002;25:210-224.

79. Godolphin W. Shared decision-making. Healthc Q 2009;12 Spec No Patient:e186-e190.

80. Bate L, Hutchinson A, Underhill J, Maskrey N. How clinical decisions are made. Br J Clin Pharmacol 2012;74:614-620.

81. Barry MJ, Edgman-Levitan S. Shared decision making--pinnacle of patient-centered care. N Engl J Med 2012;366:780-781.

82. Oshima Lee E, Emanuel EJ. Shared decision making to improve care and reduce costs. N Engl J Med 2013;368:6-8.

83. ter Meulen RH. The ethical basis of the precautionary principle in health care decision making. Toxicol Appl Pharmacol 2005;207(2 Suppl):663-667.

84. Tannahill A. Beyond evidence--to ethics: a decision-making framework for health promotion, public health and health improvement. Health Promot Int 2008;23:380-390.

85. Berger JT, DeRenzo EG, Schwartz J. Surrogate decision making: reconciling ethical theory and clinical practice. Ann Intern Med 2008;149:48-53.

86. Kass NE. Early phase clinical trials: communicating the uncertainties of 'magnitude of benefit' and 'likelihood of benefit'. Clin Trials 2008;5:627-629; discussion 630.

87. Légaré F, Brouillette MH. Shared decision-making in the context of menopausal health: where do we stand? Maturitas 2009;63:169-175.

88. Légaré F, Moher D, Elwyn G, LeBlanc A, Gravel K. Instruments to assess the perception of physicians in the decision-making process of specific clinical encounters: a systematic review. BMC Med Inform Decis Mak 2007;7:30.

89. Stacey D, Samant R, Bennett C. Decision making in oncology: a review of patient decision aids to support patient participation. CA Cancer J Clin 2008;58:293-304.

90. Elwyn G, Stiel M, Durand MA, Boivin J. The design of patient decision support interventions: addressing the theory-practice gap. J Eval Clin Pract 2011;17:565-574.

91. O'Connor A. Using patient decision aids to promote evidence-based decision making. ACP J Club 2001;135:A11-A12.

92. O'Connor AM. Using decision aids to help patients navigate the "grey zone" of medical decision-making. CMAJ 2007;176:1597-1598.

93. Roshanov PS, Fernandes N, Wilczynski JM, Hemens BJ, You JJ, Handler SM, et al. Features of effective computerised clinical decision support systems: meta-regression of 162 randomised trials. BMJ 2013;346:f657.

94. O'Connor AM, Bennett C, Stacey D, Barry MJ, Col NF, Eden KB, et al. Do patient decision aids meet effectiveness criteria of the international patient decision aid standards collaboration? A systematic review and meta-analysis. Med Decis Making 2007;27:554-574.

95. Liu J, Wyatt JC, Altman DG. Decision tools in health care: focus on the problem, not the solution. BMC Med Inform Decis Mak 2006;6:4.

96. Chen T, Wang Y, Chen H, Marder K, Zeng D. Targeted local support vector machine for age-dependent classification. J Am Stat Assoc 2014;109:1174-1187.

97. Breiman L. Random forests. Machine Learning 2001;45;5-32.

98. Hinton GE, Osindero S, The YW. A fast learning algorithm for deep belief nets.Neural Comput

2006;18:1527-1554

Appendix 1. Summary tables of the definition-related sentences about decision analysis (DA) and clinical decision analysis (CDA)

	Sources References*	the related sentences
Decision Analysis (DA)	A01 (1986)	DA -an explicit, normative and analytic approach to making decisions under uncertainty- provides a probabilistic framework for exploring difficult problems in non-deterministic domains.
	A02 (1995)	DA is the application of explicit, quantitative methods to analyze decisions under conditions of uncertainty.
	A03 (2004)	DA formalizes the decision process, highlights the factors that influence the decision, and applies mathematical rigour to quantify decision-making.
Clinical Decision Analysis (CDA)	A04 (1995)	CDA seeks to identify the optimal management strategy by modelling the uncertainty and risks entailed in the diagnosis, natural history, and treatment of a particular problem or disorder.
	A05 (1989)	CDA is a systematic method for making wise choices under just such circumstances.
	A06 (1999)	(C)DA in a quantitative approach for dealing with the uncertainties inherent in many medical decisions, including decisions about genetic testing.
	A07 (2001)	(C)DA is a quantitative by an ever increasing number of costly and confusing application of probability and utility theory to decision diagnostic tests and therapeutic interventions, decision making under conditions of uncertainty.

	A08 (2002)	(C)DA is a quantitative approach to decision making under conditions of uncertainty that can be applied to specific types of clinical problems.
	A09 (2002)	CDA is a process whereby different treatment options are assessed systematically.
	A10 (2005)	(C)DA is a formal, mathematical approach to analyzing difficult decisions faced by clinical decision makers (i.e. patients, clinicians, policy-makers).
	A11 (2006)	(C)DA is a formal, quantitative method for systematically comparing the benefits and harms of alternative clinical strategies under circumstances of uncertainty.
	A12 (2008)	(C)DA is a tool that allows users to apply evidence-based medicine to make informed and objective clinical decisions when faced with complex situations.
	A13 (2008)	(C)DA is a simulation, model-based research technique in which investigators combine information from a variety of sources to create a mathematical model representing a clinical decision.
	A14 (2009)	CDA – the application of DA to a clinical or patient-based setting – is a technique that incorporates literature-derived probabilities with expert and patient preferences to result in an informed clinical decision.

* Reference number (publication year)

A01. Wong JB, Moskowitz AJ, Pauker SG. Clinical decision analysis using microcomputers. A case of coexistent hepatocellular carcinoma and abdominal aortic aneurysm. West J Med 1986;145:805-815.

A02. Richardson WS, Detsky AS. Users' guides to the medical literature. VII. How to use a clinical decision analysis. A. Are the results of the study valid? Evidence-Based Medicine Working Group. JAMA 1995;273:1292-1295.

A03. Sonnenberg A. Patient-physician discordance about benefits and risks in gastroenterology decision-making. Aliment Pharmacol Ther 2004;19:1247-1253.

A04. Doan A, Haddawy P, Kahn CE Jr. Decision-theoretic refinement planning: a new method for clinical decision analysis. Proc Annu Symp Comput Appl Med Care 1995:299-303.

A05. Watts NT. Clinical decision analysis. Phys Ther 1989;69:569-576.

A06. McConnell LM, Goldstein MK. The application of medical decision analysis to genetic testing: an introduction. Genet Test 1999;3:65-70.

A07. Sarasin FP. Decision analysis and its application in clinical medicine. Eur J Obstet Gynecol Reprod Biol 2001;94:172-179.

A08. Burd RS, Sonnenberg FA. Decision analysis: a basic overview for the pediatric surgeon. Semin Pediatr Surg 2002;11:46-54.

A09. Manarey CR, Westerberg BD, Marion SA. Clinical decision analysis in the treatment of acute otitis media in a child over 2 years of age. J Otolaryngol 2002;31:23-30.

A10. van der Velde G. Clinical decision analysis: an alternate, rigorous approach to making clinical decisions and developing treatment recommendations. J Can Chiropr Assoc 2005;49:258-263.

A11. Elkin EB, Vickers AJ, Kattan MW. Primer: using decision analysis to improve clinical decision making in urology. Nat Clin Pract Urol 2006;3:439-448.

A12. Aleem IS, Schemitsch EH, Hanson BP. What is a clinical decision analysis study? Indian J Orthop

2008;42:137-139.

A13. O'Brien SH. Decision analysis in pediatric hematology. Pediatr Clin North Am 2008;55:287-304.

A14. Aleem IS, Jalal H, Aleem IS, Sheikh AA, Bhandari M. Clinical decision analysis: Incorporating the evidence with patient preferences. Patient Prefer Adherence 2009;3:21-24.

Appendix 2. Some articles referred to conducting the clinical decision analysis using decision tree method.

Epidemiology	Domain	the related articles
Basic	Threshold	B01 (1980), B02 (1985)
	Prognostic factor	B03 (1999)
	Cost factor	B04 (2006)
	Genetic counselling	B05 (1977)
Clinical	Screening test	B06 (1994)
	Diagnostic test	B07 (2006), B08 (2012)
	Procedures	B09 (1992), B10 (1993),<br>B11 (2001), B12 (2012), B13 (2014)
	Drug	B14 (2004), B15 (2005)

* Reference number (publication year)

B01. Pauker SG, Kassirer JP. The threshold approach to clinical decision making. N Engl J Med 1980;302:1109-1117.

B02. Harber P, Rappaport S. Clinical decision analysis in occupational medicine. Choosing the optimal FEV1 criterion for diagnosing occupational asthma. J Occup Med 1985;27:651-658.

B03. Gando S, Nanzaki S, Kemmotsu O. Disseminated intravascular coagulation and sustained systemic inflammatory response syndrome predict organ dysfunctions after trauma: application of clinical decision analysis. Ann Surg 1999;229:121-127.

B04. Connock M, Frew E, Evans BW, Bryan S, Cummins C, Fry-Smith A, et al. The clinical effectiveness

and cost-effectiveness of newer drugs for children with epilepsy. A systematic review. Health Technol Assess 2006;10(7):1-118.

B05. Pauker SP, Pauker SG. Prenatal diagnosis: a directive approach to genetic counseling using decision analysis. Yale J Biol Med 1977;50:275-289.

B06. Krahn MD, Mahoney JE, Eckman MH, Trachtenberg J, Pauker SG, Detsky AS. Screening for prostate cancer. A decision analytic view. JAMA 1994;272:773-780.

B07. Mol BW, Verhagen TE, Hendriks DJ, Collins JA, Coomarasamy A, Opmeer BC, et al. Value of ovarian reserve testing before IVF: a clinical decision analysis. Hum Reprod 2006;21:1816-1823.

B08. Burch J, Hinde S, Palmer S, Beyer F, Minton J, Marson A, et al. The clinical effectiveness and cost-effectiveness of technologies used to visualise the seizure focus in people with refractory epilepsy being considered for surgery: a systematic review and decision-analytical model. Health Technol Assess 2012;16:1-157.

B09. Johnson N, Lilford RJ, Jones SE, McKenzie L, Billingsley P, Songane FF. Using decision analysis to calculate the optimum treatment for microinvasive cervical cancer. Br J Cancer 1992;65:717-722.

B10. Gariepy AM, Creinin MD, Schwarz EB, Smith KJ. Reliability of laparoscopic compared with hysteroscopic sterilization at 1 year: a decision analysis. Obstet Gynecol 2011;118(2 Pt 1):273-279.

B11. Aoki N, Beck JR, Kitahara T, Ohbu S, Soma K, Ohwada T, et al. Reanalysis of unruptured intracranial aneurysm management: effect of a new international study on the threshold probabilities. Med Decis Making 2001;21:87-96.

B12. Liu D, Lehmann HP, Frick KD, Carter HB. Active surveillance versus surgery for low risk prostate cancer: a clinical decision analysis. J Urol 2012;187:1241-1246.

B13. Willis SR, Ahmed HU, Moore CM, Donaldson I, Emberton M, Miners AH, et al. Multiparametric MRI followed by targeted prostate biopsy for men with suspected prostate cancer: a clinical decision analysis. BMJ Open. 2014;4:e004895.

B14. Minelli C, Abrams KR, Sutton AJ, Cooper NJ. Benefits and harms associated with hormone replacement therapy: clinical decision analysis. BMJ 2004;328:371.

B15. Perreault S, Levinton C, Laurier C, Moride Y, Ste-Marie LG, Crott R. Validation of a decision model for preventive pharmacological strategies in postmenopausal women. Eur J Epidemiol 2005;20:89-101.

<한글 초록>

임상결정분석(CDA)은 임상문제의 복잡성과 불확실성을 극복하고자 개발되었다. 즉 CDA는 의료인으로서 근거바탕의 진료를 할 수 있도록 임상결정을 도와주는 도구인 것이다. 결정나무를 활용한 CDA는 6단계로 진행되며, 이의 유용성과 제한점을 살펴보았다. CDA로 얻어진 결과는 환자의 선호도와 함께 고려하여 최종 결정을 해야 할 것이다.

중심어: 결정분석, 임상결정, 결정도구, 근거중심의학
